# Preparing humanitarians to address ethical problems

**DOI:** 10.1186/s13031-020-00319-4

**Published:** 2020-11-04

**Authors:** Catherine R. McGowan, Louisa Baxter, Marc DuBois, Julian Sheather, Ruma Khondaker, Rachael Cummings, Kevin Watkins

**Affiliations:** 1 Humanitarian Public Health Technical Unit, Save the Children UK, 1 St John’s Lane, London, EC1M 4AR UK; 2 Department of Public Health, Environments & Society, London School of Hygiene & Tropical Medicine, 15-17 Tavistock Place, London, WC1H 9SH UK; 3 Department of Development Studies, SOAS University of London, 10 Thornhaugh Street, Russell Square, London, WC1H 0XG UK; 4 Médecins Sans Frontières/Doctors Without Borders (MSF), Lower Ground Floor, Chancery Exchange, 10 Furnival Street, London, EC4A 1AB UK; 5 Mental Health and Psychosocial Support, Save the Children Bangladesh, Rohingya Response, Cox’s Bazaar, Bangladesh; 6 Chief Executive Officer, Save the Children UK, 1 St John’s Lane, London, EC1M 4AR UK

## Abstract

Infectious disease outbreaks represent potentially catastrophic threats to those affected by humanitarian crises. High transmissibility, crowded living conditions, widespread co-morbidities, and a lack of intensive care capacity may amplify the effects of the outbreak on already vulnerable populations and present humanitarian actors with intense ethical problems. We argue that there are significant and troubling gaps in ethical awareness at the level of humanitarian praxis. Though some ethical guidance does exist most of it is directed at public health experts and fails to speak to the day-to-day ethical challenges confronted by frontline humanitarians. In responding to infectious disease outbreaks humanitarian workers are likely to grapple with complex dilemmas opening the door to moral distress and burnout.

## Background

Several features of infectious disease outbreaks in humanitarian contexts may produce swift, disruptive, and fatal crises. These features include high transmissibility, widespread co-morbidities, and a lack of intensive care capacity [1]. Humanitarian health care workers, like their counterparts in national health systems, are at risk of becoming infected in the course of their work. Staff illness may both reduce the capacity of a health response and dissuade others from providing support. Stigma surrounding infectious diseases may pose a challenge to community acceptance of, and involvement in, a health response. Background risks, such as crowded living conditions, high levels of malnutrition and restricted access to clean water, sanitation, and health care reinforce the threats posed by infectious diseases. Humanitarian responses to outbreaks are often implemented within other pre-existing crises such as those directly caused, or otherwise intensified by, political instability, civil unrest, armed conflict, religious intolerance, and environmental catastrophes which themselves may represent a severe and persistent threat to life. This compounding of emergencies inevitably places humanitarians and their organisations at risk and can generate unintended harms both within the health care workforce and amongst affected populations. As a result, along with critical operational challenges, humanitarian organisations are confronting a range of intense ethical problems. For example, is it ethically justified to establish and operate COVID-19 isolation and treatment centres when the supply chain for medical oxygen is unreliable, and where ventilators are in limited supply? Is it right to establish infectious disease response facilities in contexts where referral options for specialised life-saving care are limited, with the result that communities themselves will have to manage critical cases, including end-of-life provision? Faced with deadly diseases is it right for national and international staff to enjoy differential and unequal access to health care in the event of sickness? We define these and associated questions as ethical challenges because there is no response to these problems that does not directly, or indirectly, create harm. These types of ethical dilemmas are increasingly associated with moral distress and burnout amongst humanitarian actors, including those working close to outbreaks.

In 2018, the United States Agency for International Development’s Bureau for Humanitarian Assistance (formerly the Office of U.S. Foreign Disaster Assistance) funded Save the Children to lead a three-year initiative to augment global capacity to respond to major disease outbreaks. Through a consortium of partners, the READY initiative supplements existing efforts to strengthen global public health outbreak response structures and bolster the capacity of operational organisations responding to outbreaks [2]. As part of the READY initiative we conducted a review of the literature describing the ethical issues arising within humanitarian responses to infectious disease outbreaks. This line of enquiry has emerged from recent internal evaluations at Save the Children UK - including evaluations of our Ebola, yellow fever, and cholera responses - which have demonstrated concerns about moral distress, and an appetite for practical ethics guidance for field-based staff responding to infectious disease outbreaks.

Our review found significant and troubling gaps in ethical awareness at the level of humanitarian praxis including: gaps in experience with ethical deliberation, gaps in the sector’s capacity to make use of existing ethical guidance, and insufficient integration of ethics within organisational culture. Although ethical guidance for specific disease outbreaks exists, the review suggested that much of it is directed at public health experts and fails to speak to the day-to-day ethical challenges confronted by frontline humanitarians. Our review also indicated that the most relevant literature on ethics and outbreak response is: (a) recent (i.e. post-2005), (b) unevenly developed for, or often non-specific to, outbreak response (i.e. clustered around topics such as research ethics or legal issues such as quarantine), and/or (c) concentrated on a few specific diseases (such as influenza or Ebola) and not on infectious disease outbreaks more generally. We describe below the ethics gap in the humanitarian landscape, provide recommendations for providing ethics guidance for frontline humanitarian workers, and propose four complementary approaches for the sector to address the ethics gap.

## The ethics gap in the humanitarian landscape

Our review identified several areas of ethical concern that receive insufficient attention. First, humanitarian responses are not necessarily designed as public health interventions but may instead focus on the more immediate requirements of the affected population. These concerns may not align with a public health approach. For example, food distribution programmes may meet an immediate need but can result in the congregation of large numbers of people, heightening the risk of transmission [3]. The humanitarian endeavour is characterised by urgency, by a focus on the most immediate needs of a particular population (which may not correspond to the total population at risk). Humanitarian responses may be wholly or in part defined by donor interests, the needs as defined by the affected population, or on the programming priorities of national governments. When humanitarian responses do not align with public health priorities it is difficult to determine right action. Understanding the myriad of ethical quandries in this context is complicated and few humanitarian organisations recognise or respond to these fundamental differences in perspective. These agencies often lack the capacity to manage public health responses to infectious diseases and the inevitable political tensions they generate.

Second, there are many gaps in our understanding of how infectious diseases can be managed in the context of global justice and the ethics of global health. Contemporary global health inequalities are not naturally occurring phenomena. They are, directly and indirectly, the outcome of human choices and actions. They have a socio-political history deeply interwoven with, and profoundly marked by colonialism, imperialism, and racism -- processes that have led, in part, to the creation and ongoing need for international NGOs. Responding to infectious disease outbreaks thus requires sensitivity to power dynamics, the flow of goods between resource-rich and resource-poor settings, and the requirement to show respect for individual dignity and local culture [4]. Important questions include whether higher-income countries owe public health-related duties to their lower-income counterparts as a matter of distributive justice, whether former colonial powers owe reparations in recognition of the role of colonialism in creating vulnerabilities, and how these obligations might be fulfilled, particularly given calls in many higher income countries to prioritise their own national outbreak responses [5, 6]. Humanitarian workers are often distressed as they come face-to-face with the realities and injustices of lower-income countries having to rely on assistance from wealthier countries. To illustrate, Ebola elicited an international response principally because of the highly contagious nature of the disease and its ability to cross North-South boundaries, thus threatening to harm wealthy nations [7]. Other infectious diseases that have less tendency to cross borders – cholera or typhoid, for example -- threaten millions of lives but attract comparatively little attention [8].

Third, though there is no shortage of ‘humanitarian ethics guidance’ there is a paucity of practical ethical guidance aimed at the front-line humanitarian worker.

## Ethics guidance for frontline humanitarian workers

Humanitarian action generates ethical issues as routinely as logistical ones. Yet beyond the core humanitarian principles (i.e. humanity, neutrality, impartiality, and independence) the sector rarely engages with them, even where they permeate strategic and operational choices and the day to day activities of frontline humanitarians. Research shows that humanitarians are often unaware that some of the dilemmas they face require ethical deliberation, and that ethics training and support could be a resource for them [9]. Typical humanitarian management training covers the humanitarian principles but not ethics in relation to operational decision-making, and aid agencies produce hundreds of guidelines on all components of programming but have produced few ethics guidelines; ethics guidelines that do exist typically come from academics who lack an understanding of the lived reality of frontline humanitarian workers let alone frontline staff working in the context of an infectious disease outbreak. For example, the 245-page Médecins Sans Frontières (MSF) guidelines on cholera outbreak management do not contain a single reference to ‘ethics’ (nor ‘dignity’, ‘justice’ or ‘beneficence’) [10]. The guidelines are purely technical, despite decades of experience of working with ethical issues arising during cholera outbreak responses, and despite considerable focus on medical ethics among frontline staff. Furthermore, there is a lack of willingness amongst humanitarian organisations (or other authorities) to address certain operational choices as normative in the sense that they represent value judgements and are thus based neither on operational experience nor empirical facts [11]. This can create a culture where emergency assistance – the requirement to act swiftly and decisively – is at odds with ethical choices or the need for ethical deliberation. This conflict can contribute to burn-out and moral distress, i.e. the anguish and psychological instability precipitated by understanding the correct moral action to take yet being prevented from acting by institutional barriers or priorities [12]. Though the literature from the past 10–15 years shows evidence of increasing attention to the experience of moral distress amongst frontline humanitarian staff [9], much uncertainty about how to address it remains.

The relatively underdeveloped nature of practical ethical guidance for front-line humanitarian workers reflects the authors’ experience that intervention-oriented ethical reflection is uncommon within the humanitarian sector. Infectious disease outbreaks, whilst commonplace in many humanitarian settings, immerse the frontline humanitarian worker in a morass of new and amplified ethical challenges for which the literature has little to offer. We have identified three main shortcomings in the literature and how these can be addressed.

### Ethical guidance must be accessible and appropriate

Whilst there is a large and growing body of predominantly academically-driven, high-level ethical reflection on outbreak responses, practical ethical guidance for frontline humanitarian staff is in short supply, particularly for those who do not have a medical background and whose professional training is less likely to include ethics. Although we recognise the critical importance of high-level principles, it is less obvious how they should be interpreted and applied in the fast-moving, highly pressured and often confused context of an infectious disease outbreak. Additionally, the extant literature often describes the experience of international rather than national staff (who are more likely to be working on the front lines in positions that place them at heightened risk). Moreover, much of the available guidance blurs the distinction between the ethics of the possible and best practice, producing lists of ethical practice that appear unattainable in the difficult circumstances of humanitarian response [13]. Perversely this can increase moral distress amongst humanitarian workers and, by extension, undermine the delivery of much needed services to affected populations.

### Research scope should be broadened

There is little in the way of sound research on the real-world ethical problems faced by humanitarian organisations or frontline humanitarian staff; though there are some notably useful exceptions [13–17] including real-world cases presented as the basis for ethical reflection drawing on the experience of humanitarians in Syria [16], and amongst frontline staff deployed with MSF and the International Rescue Committee [16, 17]. Much of what has been published is narrowly based on the experience of a few international staff from Western or high-resource health systems. For example, Hunt et al. developed an ethics framework for health practice, the collective basis for which amounted to 45 expatriate clinicians; left out were non-medical staff, national staff of all professions, and decision-making and programming staff [18]. Furthermore, there is almost no literature addressing the real-world ethical problems inherent in infectious disease outbreaks.

### Practical ethical guidelines need to be evaluated

There are few published evaluations evidencing the utility of practical ethical guidance in real world settings, and none specific to infectious disease outbreaks [16]. Research into how organisations both prepare and support humanitarian health responders is needed, as well as an understanding of how to embed ethical deliberation in existing agency processes, analysis, and culture.

## Organisational responsibilities in dealing with ethical tensions

The invisibility of practical ethics has consequences; it reduces the capacity of the sector to absorb ethical guidance because it is foreign to its structures, leadership, and personnel (aside from some medical professionals and some other professional groups). The situation does, however, offer opportunities to reduce the likelihood of moral distress among humanitarian workers by making existing ethical guidelines more accessible, by improving the sector’s capacity for ethical reflection, and by providing practical guidance relevant to real-world ethical problems such as those typically encountered in infectious disease outbreaks.

We recommend the four following complementary approaches for the sector to address the ethical gap: 1) foster a culture of ethical deliberation and compromise, 2) provide institutional support to all staff including training specifically geared to the practical realities of an infectious disease outbreak, 3) use decision-making tools, and 4) support staff in moral distress.

### Foster a culture of deliberation and compromise: process matters

Few ethical choices in outbreak response can be understood, or resolved, by reference to a universal equation or standard formula. Principles tend toward the high-level and the abstract and can, as Hugo Slim concludes, result in humanitarian workers feeling that “[they] always tell us what is good to do but they do not easily tell us what is best to do in difficult situations” [19 p. 44]. Conflict between principles and realities where all choices lead to some harm are inevitable [19] and practitioners need to understand this. Organisations should acknowledge that the application of principles to real world situations requires experience, self-reflection, and interpretation [19]. The misperception underlying much of the discourse on ethics is that deliberation should yield a triumphant claim (via reason, agreement, or authority) – a ‘right’ answer or way forward. Instead, deliberation should consider how the various options bring benefits and harms/costs and what, if any, compromise is possible [20]. This acknowledges the importance of the *process* by which decisions are made – and hence the imperative of procedural ethics [11, 21]. Procedural ethics aims to improve the quality and consistency of decisions, as well as reducing the distress and frustration of those affected by processes lacking ethical sensitivity.

### Provide institutional ethics support and guidance

There is risk to staff who are not properly prepared and supported in their work. Both primary and vicarious trauma may cause significant distress. Organisational support is, therefore, not optional. Agencies have a duty of care to anticipate risks of moral distress, to establish a transparent feedback mechanism to understand staff experience of such risks, and to manage or mitigate against them. The principle of reciprocity, linked to fairness, increases the obligation to protect responders from harm because they accept heightened risks as part of their work. There are several actions and stances that the organisation can take to meet this obligation:

- **Signal the importance of ethics** by devoting resources to addressing the main ethical issues facing the organisation and its staff and articulating the organisation’s ethical and humanitarian principles. Ideally, training in ethics (and rational choice theory) would be integrated into humanitarian training more broadly.
- **Demonstrate understanding of how positionality affects ethical considerations.** Recognise and destabilise the overwhelming whiteness, and maleness, of the debate on humanitarian ethics and allow space for perspectives from the global south [22]. Address racism and sexism as ethical issues.
- **Recruit the right people.** Just as recruitment of staff should ensure the necessary professional competence, so it must seek those with experience and understanding of ethics and an appropriate personal profile for working in ethically compromising circumstances. In addition, staff who have experience working in infectious disease outbreaks are likely to have a better understanding of the associated risks, both to themselves and to affected populations, and may be better able to engage in suitably informed ethical decision-making.
- **Prepare staff in advance for what they are likely to encounter.** Support should begin at the pre-response stage, allowing staff to, “carefully consider whether they are prepared to deal with ethical issues that may lead to moral and psychological distress” [23]. International aid workers need a pre-departure briefing on expected conditions [24]. This should include discussion of their own beliefs, values, assumptions and biases, as well as relevant local beliefs and values [15].
- **Shield frontline staff from having to make challenging individual or ad hoc decisions** where these can be anticipated and preempted by action at a higher level: “[h] aving clear rules and guidelines for responders may decrease the moral stress that they take on individually” [25, p. 54]. Clear rules and priorities must also be set where there are differential policies for local or international staff, or between staff and patients [25, 26].
- **Provide ethical clarity particularly for politically charged interventions** (surveillance, coercive restrictions), circumstances characterised by abuse and violence by authorities, and services to marginalised communities such as ethnic minorities, indigenous communities, or displaced persons.
- **Recognise and address vulnerabilities** related to age, gender, and disability.
- **Foster team support.** Team support is critical to mental health generally and when dealing with moral distress in particular [27, 28]; in simple terms, talking to others helps. This should occur organically, and be supported by the agency (e.g. regular team debriefings to include ethical dilemmas), as should deliberate mentoring [15]. Research in healthcare settings shows that structured discussions of ethics can help distinguish between unavoidable harm and ethical failing [9]. It can, however, be challenging to make space for this in an emergency response, particularly in an outbreak setting in which infection prevention and control measures may complicate face-to-face interaction [29].
- **Encourage personal responsibility and self-care.** Agencies should foster a culture in which staff look after themselves [19]. Among the essential practical virtues for humanitarian workers are certain habits of self-care, the capacity to engage in meaningful self-reflection and the willingness to interrogate their own values.
- **Use scenario or case-based training**, particularly that which is informed by local knowledge [16, 23]. Evidence suggests that amongst frontline humanitarian staff case-based training is more effective than abstract ethics lectures [15]. Scenario-based training, particularly that which is informed by local knowledge and contextualised through community involvement, also reinforces the need to seek support for ethical issues and overcome them as a team [15].
- **Document and share ethically challenging cases and promote institutional memory** to assist when similar situations occur again [17].
- **Engage expert support on ethics to assist when necessary** [13].

### Use decision-making tools and frameworks

Frameworks or tools exist to help navigate ethical problems. The ethics literature consistently refers to principles of decision-making procedure that draw upon the work of Daniels and others [11, 21, 30]. This framework includes five key values: accountability, inclusiveness, openness and transparency, reasonableness, and reviewability. The Humanitarian Health Ethics research group has produced a six-step tool [20] that helps identify ethical issues and the related costs and benefits of options. Whilst generally seen as helpful in tests, it raised concerns about the requirement for time or prior familiarity [9]. A second framework was produced by Clarinval and Biller-Adorno, and involves a similar, though arguably more challenging, process [13]. These tools translate operational or programmatic dilemmas/challenges into the language of ethics, helping to clarify what is at stake. They also bring structure and consistency to decisions [31]. Ultimately, tools and guidelines need to be immediately relevant to be useful; hence, tools and guidelines which speak directly to the types of challenges inherent in infectious disease outbreaks are needed. Furthermore, there is a need to evaluate and improve the utility of exiting frameworks and tools.

### Support staff in moral distress

Moral distress can be precipitated by individual, institutional, and broader external factors and has consequences for the psychological, emotional, and physical health of staff, and for the care they deliver [32, 33]. The need to address moral distress is both a duty (insofar as it fulfils basic obligations of organisations to provide for the occupational health of their staff) and highly pragmatic. Just as with health or security risks, “certain issues faced by humanitarian aid workers are ethical issues, not geopolitical or managerial questions, and humanitarian actors are therefore likely to face ethical dilemmas that lead to moral distress” [13, p. 5]. Moral distress forms part of a humanitarian’s *moral experience*, even if not all moral distress is the direct result of ethical tension or ethical lapses. This experience exceeds the rational or deliberative processing of ethical tensions, as it is rooted in individual psychology rather than within a particular context [29]. Dealing with moral distress forms part of a wider organisational duty to support the overall mental health and well-being of humanitarian aid workers [9].

## Conclusion

Humanitarian workers responding to infectious disease outbreaks grapple with ethical dilemmas of a novel scale and severity, opening the door to moral distress and burnout. Yet ethics and ethical deliberation occupy little turf in the humanitarian enterprise. Our review revealed a significant discrepancy between ethical guidance for disease outbreaks at a theoretical or macro level (which has notably expanded over the past decade), and guidance designed for international frontline staff (particularly frontline staff who do not have a medical background). Ultimately, though ethical guidance specific to infectious disease outbreaks exists, it is largely disconnected from the capacity of the humanitarian agency.

Finally, it is important to acknowledge that the ethics gap in humanitarian response is not simply a technical hole to be filled. Education and training on ethics is important, yet the gap involves structural issues that cannot be resolved solely by guidance or capacity building.

As humanitarian organisations scramble to deliver urgent assistance and protection in the COVID-19 pandemic, long lists of ethical guidelines and professional or academic deliberation may prove paralytic to overworked field teams or inadvertently increase stress over making the right choices. That leaves the leadership of humanitarian agencies to ask themselves: what are the moral values that we seek to fulfil in the course of responding to infectious disease outbreaks, and how do we translate these into our strategic, programmatic, and individual choices?

## Data Availability

Not applicable.
